# Genotype–phenotype correlates in Joubert syndrome: A review

**DOI:** 10.1002/ajmg.c.31963

**Published:** 2022-03-03

**Authors:** Simone Gana, Valentina Serpieri, Enza Maria Valente

**Affiliations:** ^1^ Neurogenetics Research Center IRCCS Mondino Foundation Pavia Italy; ^2^ Department of Molecular Medicine University of Pavia Pavia Italy

**Keywords:** ciliopathies, genotype–phenotype correlations, Joubert syndrome, primary cilia, pleiotropy

## Abstract

Joubert syndrome (JS) is a genetically heterogeneous primary ciliopathy characterized by a pathognomonic cerebellar and brainstem malformation, the “molar tooth sign,” and variable organ involvement. Over 40 causative genes have been identified to date, explaining up to 94% of cases. To date, gene‐phenotype correlates have been delineated only for a handful of genes, directly translating into improved counseling and clinical care. For instance, JS individuals harboring pathogenic variants in *TMEM67* have a significantly higher risk of liver fibrosis, while pathogenic variants in *NPHP1*, *RPGRIP1L*, and *TMEM237* are frequently associated to JS with renal involvement, requiring a closer monitoring of liver parameters, or renal functioning. On the other hand, individuals with causal variants in the *CEP290* or *AHI1* need a closer surveillance for retinal dystrophy and, in case of *CEP290*, also for chronic kidney disease. These examples highlight how an accurate description of the range of clinical symptoms associated with defects in each causative gene, including the rare ones, would better address prognosis and help guiding a personalized management. This review proposes to address this issue by assessing the available literature, to confirm known, as well as to propose rare gene‐phenotype correlates in JS.

## 1 INTRODUCTION

Joubert syndrome (JS) is a rare congenital neurodevelopmental primary ciliopathy with a population‐based prevalence reaching 1.7 per 100,000 in the age range 0–19 years (Nuovo et al., 2020). First described by Dr Marie Joubert about 50 years ago (Joubert, Eisenring, Robb, & Andermann, 1969), JS is now diagnosed upon recognition of a pathognomonic malformation of the midbrain–hindbrain junction which results in the brain imaging finding “molar tooth sign” (MTS). This malformation, found in all patients, consists of cerebellar hypoplasia with vermian dysplasia, thick and horizontally oriented superior cerebellar peduncles, and an abnormally deep interpeduncular fossa (Maria et al., 1997). A spectrum of severity of the MTS has been reported (Poretti, Huisman, Scheer, & Boltshauser, 2011), and mild MTS presentations may be difficult to assess. Conversely, other cerebellar and brainstem malformations are sometimes wrongly interpreted as a mild MTS, leading to misdiagnosis (Aldinger et al., 2014; D'Abrusco et al., 2021; Powell et al., 2021).

The clinical picture is evident from neonatal age with hypotonia, abnormal eye movements (mainly ocular motor apraxia, OMA), developmental delay and, in a subset of patients, episodic breathing dysregulation; later clinical signs comprise cerebellar ataxia and, frequently, cognitive impairment (Brancati, Dallapiccola, & Valente, 2010; Doherty, 2009; Romani, Micalizzi, & Valente, 2013). Even though some facial dysmorphisms are often observed, facial features do not strongly support the clinical diagnosis, as in many patients they can be unremarkable (Braddock, Henley, & Maria, 2007). A variable involvement of other organs (such as eye, kidney, liver, and skeleton) is present in two‐thirds of individuals with JS, and can manifest at different ages and with variable severity (Bachmann‐Gagescu et al., 2015). This complex presentation makes JS a multisystem condition, and some clinical issues may be progressive, complicating medical management.

Based on the presence of associated extra‐CNS features, JS can be classified into clinical subgroups (Brancati et al., 2010; Romani et al., 2013):

- Purely neurological JS (pure JS)
- JS with ocular involvement (JS‐O)
- JS with renal involvement (JS‐R)
- JS with oculorenal involvement (JS‐OR)
- JS with hepatic involvement (JS‐H, or COACH syndrome)
- JS with orofaciodigital involvement (JS‐OFD, or OFDVI syndrome)
- JS with acrocallosal features
- JS with Jeune asphyxiating thoracic dystrophy

Occasional features observed in all subgroups include polydactyly, which can be pre‐, meso‐, or post‐axial and variably involve hands and feet, and other CNS abnormalities, such as corpus callosum abnormalities, hydrocephalus, encephalocele, or polymicrogyria.

While these complex phenotypes had initially been termed “Joubert syndrome related disorders” (Gleeson et al., 2004; Satran, Pierpont, & Dobyns, 1999), nowadays the unifying term “JS” applies to all patients with the MTS, including those with and without any extra‐neurological involvement (Romani et al., 2013).

Like other syndromic ciliopathies, JS is characterized by extreme genetic heterogeneity with more than 40 causative genes, all of which encoding proteins responsible for the formation or functioning of the primary cilium, a subcellular organelle playing essential roles in developing and adult tissues (Reiter & Leroux, 2017). Pathogenic variants in known genes overall account for ~62–94% of affected individuals depending on the cohort, sequencing method, and criteria for defining pathogenicity of identified variants (Bachmann‐Gagescu, Dempsey, et al., 2015; Parisi, 2019; Phelps et al., 2018; Shaheen et al., 2016). In the largest cohort reported to date, five major genes (*CPLANE1*, *CC2D2A*, *AHI1*, *CEP290*, and *TMEM67*) accounted each for ~6–9% of JS cases, three additional genes (*CSPP1*, *TMEM216*, and *INPP5E*) accounted each for ~3%, and six more genes accounted each for ~1–2%; the remaining genes were mutated only in few families (Bachmann‐Gagescu, Dempsey, et al., 2015).

JS is mainly inherited in an autosomal recessive fashion, with the exception of an X‐linked recessive form due to pathogenic variants in the *OFD1* gene (Coene et al., 2009) and a recently reported autosomal dominant form due to truncating or splice‐site variants in the *SUFU* gene (Serpieri et al., 2021). Of note, almost all JS genes have also been implicated in other ciliopathies, such as Meckel syndrome (MKS), isolated nephronophthisis (NPHP), Leber congenital amaurosis (LCA), oral‐facial‐digital syndromes (OFDS), Bardet‐Biedl syndrome (BBS), and others. While the JS diagnosis is usually made early in life in the setting of a neuropediatrics or genetics clinic (and can even be suspected prenatally), the possible impairment of other systems requires patients to enter a diagnostic workflow and regular follow‐up examinations, with referral to distinct specialists according to the organs which are involved. In this light, the establishment of gene‐phenotype correlations would enable reliable prognostic predictions and ensure the optimal assessment and management of disease complications. Yet, despite certain JS gene variants appear to be highly associated with specific features, such correlations are still challenging, due to the extreme genetic heterogeneity of this ciliopathy. Indeed, even in the largest published cohorts, the number of patients with pathogenic variants in the same gene is small, limiting statistical power for correlations.

**FIGURE 1:**
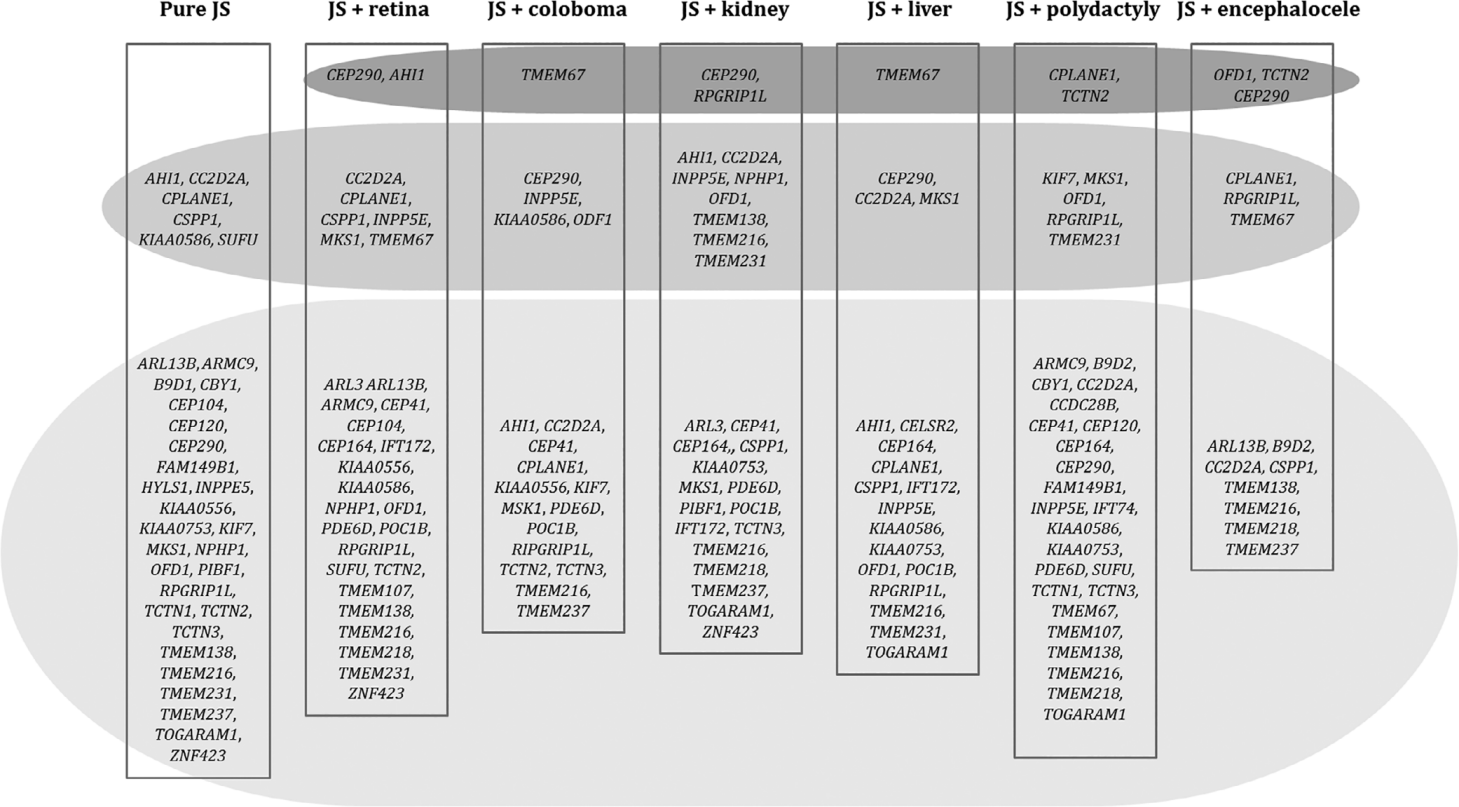
Genotype–phenotype correlations in Joubert syndrome. Top (dark gray oval): genes definitively associated with the feature (statistically significant associations as detected in large studies). Middle (medium gray oval): genes probably associated with the feature (associations reported in three or more papers or in at least 10 distinct families). Bottom (light gray oval): genes possibly associated with the feature (associations reported in less than three studies). JS: Joubert syndrome

Here, we aim to provide an overview of the more consistent and reliable correlations between phenotype and genetic cause in JS, supporting the notion that these correlates may help prognostic definition and the development of a personalized management; in addition, we mention possible associations between gene and phenotype, that still require confirmation in larger cohorts (Figure 1). Finally, in a concluding paragraph, we underline the clinical and genetic overlap of JS with other ciliopathies, in particular MKS. JS and MKS were originally described as distinct and clinically recognizable entities; however, to date, at least 16 genes have been found to cause both conditions, and the more cases are reported, the more blurred their clinical distinction becomes. Indeed, these syndromes show a variable combination of overlapping clinical findings and likely represent different expressions of the same disease spectrum.

## 2 METHODOLOGY

We performed a literature review, searching for English written publications in the National Center for Biotechnology Information's PubMed database (https://www.ncbi.nlm.nih.gov/pubmed), using the following search terms: “Joubert syndrome” AND “gene” OR “genetics.” We selected all relevant articles from 2004, when the first gene implicated in JS, *AHI1*, was identified (Ferland et al., 2004), to present. In reporting the results, emphasis has been given to larger studies and more consistent associations, while studies reporting single or few observations have been referenced but not discussed in detail.

## 3 SYSTEM‐BY‐SYSTEM REVIEW OF GENOTYPE–PHENOTYPE CORRELATION

### 3.1 Brain imaging

While essential to establish the diagnosis of JS, the MTS is not usually helpful to establish correlations with different genotypes. A possible exception is represented by the detection of thinner superior cerebellar peduncles and less severe vermis hypoplasia seen in patients with *NPHP1* homozygous deletion (Castori et al., 2005; Parisi et al., 2004). In addition, a “mild MTS” consisting in vermis hypoplasia, superior cerebellar folial dysplasia, and subtle‐to‐mild abnormalities of the superior cerebellar peduncles (which variably appeared long, thick, and horizontal), has been reported in patients with *SUFU* heterozygous loss of function variants as well as in some patients carrying biallelic variants in *CBY1*, *CPLANE1*, and *FAM149B1* (Enokizono et al., 2017; Epting et al., 2020; Serpieri et al., 2021; Shaheen et al., 2019).

Aside from the MTS, a wide range of additional brain anomalies have been described in JS individuals, such as occipital encephalocele, ventriculomegaly, dysgenesis of the corpus callosum, neural migration defects, and hypothalamic hamartoma (Bachmann‐Gagescu, Dempsey, et al., 2015; Poretti et al., 2017). All these features have been associated with variants in several genes, lacking specific gene‐phenotype correlates.

Within the large JS cohort by the University of Washington (UW), encephalocele (usually occipital) was present in 8% of individuals, significantly correlating with pathogenic variants in either *OFD1* or *TCTN2* (Bachmann‐Gagescu, Dempsey, et al., 2015). However, this malformation has been occasionally identified in patients carrying pathogenic variants in a large number of other JS genes, including all the commonly mutated ones (Ben‐Salem, Al‐Shamsi, Gleeson, Ali, & Al‐Gazali, 2014; Cantagrel et al., 2008; Edvardson et al., 2010; Gorden et al., 2008; Lee et al., 2012; Suzuki et al., 2016; Van De Weghe et al., 2021).

Poretti et al. reported neuroimaging findings of 110 JS patients, of whom 89 (81%) with a genetic diagnosis. In this series, dysgenesis of the corpus callosum was identified in patients with pathogenic variants in *CC2D2A*, *CPLANE1*, *CSPP1*, and *CELSR2* (Poretti et al., 2017). Notably, the only patient of the cohort with *KIF7*‐related JS had a normal corpus callosum, although pathogenic variants in *KIF7* are known to cause both JS and acrocallosal syndrome (ACLS), which is characterized by corpus callosum agenesis, distal anomalies of limbs, minor craniofacial anomalies, and intellectual disability (Dafinger et al., 2011; Putoux et al., 2011). In other studies, corpus callosum anomalies have been occasionally detected in association to *KIF7* as well as several other genes (Akizu et al., 2014; Bachmann‐Gagescu, Phelps, et al., 2015; Bader et al., 2016; Ben‐Salem et al., 2014; Edvardson et al., 2010; Roosing et al., 2016; Shen et al., 2020; Stephen et al., 2017; Wentzensen et al., 2016).

Migration defects described in JS patients include cortical, midbrain, and cervicomedullary heterotopias, polymicrogyria, fronto‐temporal pachigyria, and macrogyria, and have been variably reported in patients carrying pathogenic variants in *AHI1, CC2D2A, CSSP1, KIAA0586, OFD1, PIBF1, RPGRIP1L, TCTN1*, and *SUFU* (Ben‐Salem et al., 2014; De Mori et al., 2017; Dixon‐Salazar et al., 2004; Field et al., 2012; Kroes et al., 2016; Poretti et al., 2017; Shen et al., 2020; Tuz et al., 2014; Zhang et al., 2021; Zhang, Sun, Xu, Che, & Liu, 2021).

### 3.2 Neurodevelopmental features

Neurodevelopmental features in JS are related to the underlying brain malformation and, as such, represent constant features of the syndrome, and usually the earliest to manifest. These consist in neonatal hypotonia, abnormal eye movements (mainly ocular motor apraxia, OMA), and breathing dysregulation (apneas and/or hyperpneas usually resolving within few months of life). A delay of developmental milestones becomes evident soon after, followed by ataxia (with broad‐based, unsteady gait and difficulties in running or climbing stairs) and intellectual disability of variable severity; nevertheless, a minority of individuals have borderline, or even normal cognitive functions (Bulgheroni et al., 2016; Poretti, Alber, Bürki, Toelle, & Boltshauser, 2009; Summers et al., 2017). Notably, Poretti et al. reported that neuroimaging may predict the neurodevelopmental outcome, as a high degree of vermis hypoplasia was found to correlate with worse prognosis (Poretti et al., 2017). Of note, expressive speech is more affected than comprehension, also due to concurrent oral‐motor apraxia (Summers et al., 2017).

### 3.3 Neurological phenotype

Patients presenting only neurological manifestation of JS without other organ involvement are hystorically classified as “pure JS.” Overall, in the UW cohort, only 68 out of 201 exhaustively phenotyped patients (34%) had an exclusively neurological phenotype (Bachmann‐Gagescu, Dempsey, et al., 2015), eventually associated with polydactyly and other brain abnormalities (Parisi, 2019; Valente, Dallapiccola, & Bertini, 2013). Similarly, neurological JS accounted for 28% in another series of 100 JS individuals by the National Institutes of Health (NIH) Clinical Center (Vilboux et al., 2017). In contrast with these observations, a neurological phenotype was the commonest subtype (62.7%) in another, distinct cohort of 59 patients, but age at examination was too early to assess the occurrence of other organ abnormalities, hampering gene‐phenotype correlates (Radha Rama Devi, Naushad, & Lingappa, 2020).

Although no major genes have been statistically linked to this purely neurological presentation, some genes seem to recur more frequently in this group. For instance, in the UW cohort, JS patients mainly carried pathogenic variants in *CPLANE1* (13%), *CC2D2A* (13%), *AHI1* (12%), and *CSPP1* (9%) (Bachmann‐Gagescu, Dempsey, et al., 2015). In the NIH cohort, almost 29% of JS patients carried *CPLANE1* defects, while 18% and 14% had pathogenic variants in *CC2D2A* and *KIAA0586*, respectively (Brooks et al., 2018; Vilboux, Doherty, et al., 2017). An association of *CPLANE1* defects with JS was also detected in a smaller Northern European cohort of 51 JS patients, in which five out of six patients with causative variants in *CPLANE1* showed a purely neurological phenotype (Kroes et al., 2016). More significantly, in a large cohort of 313 JS individuals mainly of Italian origin, 27 out of 28 patients carrying biallelic variants in *CPLANE1* manifested an exclusively neurological phenotype, although polydactyly was a common associated feature (Romani et al., 2015). Of note, we recently reported a novel association of a relatively mild neurological phenotype with haploinsufficiency of the *SUFU* gene, which to date represents the only genetic cause of JS with dominant inheritance and reduced penetrance (Serpieri et al., 2021).

Besides these recurrent genes, this neurological presentation has been occasionally reported in association with nearly all JS genes, including those known to cause distinct JS subtypes and even other ciliopathies (such as *MKS1, AHI1, B9D1, KIF7*, or *CEP120*), challenging prognostic indications (Akizu et al., 2014; De Mori et al., 2017; Epting et al., 2020; Huppke et al., 2015; Latour et al., 2020; Lee et al., 2012; Luo et al., 2019; Oka et al., 2016; Roosing et al., 2016; Roosing, Romani, et al., 2016; Serpieri et al., 2021; Shaheen et al., 2019; Shen et al., 2020; Sivathanu, Vetrichelvan, Balakrishnan, & Manokaran, 2020; Srour et al., 2012; Van De Weghe et al., 2017) .

### 3.4 Abnormal ocular movements

Abnormal ocular movements in JS patients, which tend to improve over time, include OMA, nystagmus, and strabismus. OMA manifests as the abolition of the vestibulo‐ocular reflex, decreased smooth pursuit, and the inability to visually follow objects, which is compensated by turning head movements. In a recent comprehensive review of 254 JS individuals with different eye phenotypes, OMA was the commonest ocularmotor abnormality in JS, associated with variants in most genes (Wang et al., 2018). Of note, patients with heterozygous *SUFU* variants may present with a spectrum of neurodevelopmental phenotypes encompassing congenital OMA and mild JS (Schröder et al., 2020; Serpieri et al., 2021).

Horizontal nystagmus at birth, improving with age, is also a frequent feature; besides this, torsional and pendular rotatory nystagmus have been occasionally observed. *AHI1* molecular defects have been frequently correlated with this issue, but causative variants in several other genes have also been reported (Edvardson et al., 2010; Niceta et al., 2020; Wang et al., 2018).

Strabismus is also commonly observed in JS as in many other conditions associated with nonprogressive cerebral or cerebellar abnormalities (Salman & Chodirker, 2015). In the comprehensive review by Wang et al., *TMEM237* was the commonest altered gene in patients with this complication but, as for other ocular movement abnormalities, strabismus was also associated to several other gene variants (Huppke et al., 2015; Niceta et al., 2020; Wang et al., 2018).

### 3.5 Other neurological features

Behavioral disturbances, when present, include temper tantrums, self‐injury, autism, depression, anxiety, and auditory hallucinations (Bachmann‐Gagescu, Dempsey, et al., 2015; Bachmann‐Gagescu, Phelps, et al., 2015; Bulgheroni et al., 2016) and can have an enormous impact on the quality of life for people with JS and their families. Variability in neurocognitive functions and behavior have been reported among siblings harboring the same gene variants, suggesting that cognitive and neurobehavioral profiles in JS are not entirely related to the underlying genetic cause (Poretti et al., 2009; Poretti et al., 2017).

Seizures have been observed in more than 10% of JS individuals but no seizure type nor genetic cause appear to be prevalent (Bachmann‐Gagescu, Dempsey, et al., 2015). In the large UW cohort, none of the 55 individuals with seizures presented causative variants in *CEP290* (despite this gene represented the third commonest genetic cause of JS), suggesting a negative association (Bachmann‐Gagescu, Dempsey, et al., 2015). However, other studies have occasionally reported seizures in *CEP290*‐mutated patients (Helou et al., 2007). Conversely, biallelic variants in *CC2D2A* seem to be more commonly associated with seizures (Bachmann‐Gagescu et al., 2012). Several additional genes have been occasionally found mutated in JS patients with epilepsy, including the commonly mutated *AHI1, KIAA0586, MKS1*, and *TMEM67*, as well as many other rarely mutated genes (Bachmann‐Gagescu, Dempsey, et al., 2015; Bader et al., 2016; Dehghani et al., 2017; Inskeep et al., 2022; Kroes et al., 2016; Rafiullah et al., 2017; Sivathanu et al., 2020; Sumathipala et al., 2020; Van De Weghe et al., 2017; Wentzensen et al., 2016).

### 3.6 Eyes and vision

The ocular findings in JS range from mild to severe, often depending on the underlying genetic cause; sometimes, variability can be noted even within the same genotype. Ocular involvement can be either degenerative (e.g., retinal dystrophy) or developmental (e.g., coloboma), even if rarely co‐occurring in the same individual (Brooks et al., 2018; Wang et al., 2018).

The spectrum of ophthalmological features in JS has been prospectively ascertained in the UW and NIH cohorts, while two other studies have reported a detailed analysis of the published literature (Bachmann‐Gagescu, Dempsey, et al., 2015; Brooks et al., 2018; Vilboux, Doherty, et al., 2017; Wang et al., 2018).

Retinal involvement (defined as abnormal electroretinogram, abnormal optical coherence tomography, and/or abnormal fundus oculi with visual impairment) was present in 24–30% of individuals in the NIH and UW cohorts, while coloboma was seen in 28 and 17% patients, respectively. Consensus in the literature is that only 2–3% of patients with JS have both coloboma and retinal degeneration (Bachmann‐Gagescu, Dempsey, et al., 2015; Brooks et al., 2018).

Retinal dysfunction in JS is caused by progressive degeneration of retinal photoreceptor cells, which contain specialized primary cilia. The retinal disease can range from the extremely severe Leber congenital amaurosis (LCA) to pigmentary retinopathies evident at fundus oculi examination or milder forms of retinal dystrophy detectable only by electroretinogram, with variably conserved vision (Wang et al., 2018). This phenotype has been associated with a long list of genes, yet the two commonest players clearly are *CEP290* and *AHI1* (Bachmann‐Gagescu, Dempsey, et al., 2015; Brancati et al., 2007; Brooks et al., 2018; Chaki et al., 2012; Edvardson et al., 2010; Kar, Phadke, Das Bhowmik, & Dalal, 2018; Lambacher et al., 2016; Lee, Silhavy, Lee, et al., 2012; Powell et al., 2020; Srour et al., 2015; Suzuki et al., 2016; Valente et al, 2006; Van De Weghe et al., 2021; Wang et al., 2018). In the UW cohort, a retinal phenotype was observed in all patients with *CEP290*‐related JS, an association which remained statistically significant even after Bonferroni correction (odds ratio [OR] 22.9, confidence intervals [CI] 6.7–78.4; *p* < .0001) (Bachmann‐Gagescu, Dempsey, et al., 2015), and in eight out of 16 JS individuals with *AHI1* pathogenic variants. Similarly, in the NIH cohort, six out of seven patients with biallelic variant in *CEP290* and four out of six mutated in *AHI1* showed retinal disease (Bachmann‐Gagescu, Dempsey, et al., 2015; Brooks et al., 2018; Wang et al., 2018). In our experience, 19 of 44 patients with JS and oculorenal involvement (mainly LCA and retinitis pigmentosa) carried biallelic variants in *CEP290*, while this gene was mutated only in two out of 84 patients with other JS phenotypes. Similarly, when performing a molecular screening of the *AHI1* gene, we detected a retinal phenotype in eight out of 11 mutated patients (Brancati et al., 2007; Valente et al., 2006). Moreover, these two genes seem more likely associated with more severe retinal degeneration than others, such as *INPP5E, MKS1*, and *NPHP1* (Bachmann‐Gagescu et al., 2020). A specific association of retinal dystrophy with macular staphyloma has also been reported in few patients with *AHI1*‐ and *INPP5E*‐related JS (Toma et al., 2018), while the “morning glory disc anomaly” has been observed in an Austrian family with biallelic *TMEM237* pathogenic variants (Huang et al., 2011).

Interestingly, in the UW series, a negative correlation was observed between *TMEM67* pathogenic variants and retinal disease (OR 0.1, CI 0.01–0.8; *p* = .006), indicating that *TMEM67*‐mutated patients are less likely to be diagnosed with the retinal disease (Bachmann‐Gagescu, Dempsey, et al., 2015). Similarly, in the NIH cohort, no retinal degeneration was observed in patients with *TMEM67* defects, as well as in those carrying *CPLANE1* and *KIAA0586* variants (Vilboux, Doherty, et al., 2017), and we also reported these negative associations in our cohort (Iannicelli et al., 2010; Romani et al., 2015; Roosing et al., 2015).

Pigmentary irregularities in the peripheral or mid‐peripheral retina, or in the entire retina, which are early signs of retinal dysfunction, have been reported in 4.5% of JS patients. About 72% of them carried *AHI1* (five patients) or *CEP290* (three patients) molecular defects, but causative variants had also been detected in *CPLANE1* and *CC2D2A* (Wang et al., 2018).

Unilateral or bilateral colobomas result from abnormal closure of the optic cup during development and mostly affect the posterior segment of the eye (involving the retinal pigmented epithelium and/or the optic nerve), although choroid and iris colobomas have also been observed (Bachmann‐Gagescu, Dempsey, et al., 2015; Wang et al., 2018). The gene associated with the greatest number of patients with ocular colobomas is *TMEM67*. In two early studies, we reported chorio‐retinal or optic nerve colobomas in about 55% of patients with *TMEM67*‐related JS, all with liver fibrosis (Brancati et al., 2009; Iannicelli et al., 2010). In the UW cohort, 34 patients had colobomas and 12 of them carried mutations in *TMEM67* and also presented associated liver fibrosis, a correlation which remained statistically significant even after Bonferroni correction (OR 22.9, CIs 8.6–61.1). Similarly, in the NIH cohort, 28 patients (out of 99) had coloboma, of whom 18 with mutations in *TMEM67* (Bachmann‐Gagescu, Dempsey, et al., 2015; Brooks et al., 2018). Besides *TMEM67*, several other genes have been occasionally found mutated in association with coloboma in several independent studies (Bachmann‐Gagescu, Dempsey, et al., 2015; Brooks et al., 2018; Lee, Silhavy, Lee, et al., 2012; Niceta et al., 2020; Utsch et al., 2006; Wang et al., 2018).

Microphthalmia and/or anophthalmia, frequently accompanying colobomas, have rarely been identified in few patients with biallelic variants in *CELSR2*, *CEP120, CSPP1, PDE6D, TMEM67, TMEM138*, and *TMEM216* (Ben‐Salem et al., 2014; Edvardson et al., 2010; Kroes et al., 2016; Lee, Silhavy, Lee, et al., 2012; Powell et al., 2020; Thomas et al., 2014; Vilboux et al., 2017; Vilboux, Doherty, et al., 2017).

In JS individuals, visual function may also be occasionally impacted by optic atrophy. On examination at the NIH Clinical Center, eight out 99 patients had optic nerve atrophy (9%). Of them, three had *KIAA0586*‐ and two had *MKS1*‐related JS, the remaining patients harboring causative variants in *CC2D2A*, *INPP5E*, and *CSPP1*. Only in two individuals, optic atrophy occurred in association with retinal degeneration, respectively, in a patient with *MKS1*‐ and in a patient with *INPP5E*‐related JS (Brooks et al., 2018). Several other genes have been found mutated in additional reports of patients with optic nerve atrophy or hypoplasia (Brooks et al., 2018; Edvardson et al., 2010; Luo et al., 2021; Niceta et al., 2020; Wentzensen et al., 2016).

Ptosis can be either unilateral or bilateral with variable grade of severity. It occurred in 28 of 98 patients (29%) of JS patients assessed in the NIH cohort, with the commonest associated genes being *TMEM67* and *CPLANE1* (Brooks et al., 2018). Conversely, in a literature review of 35 patients with JS‐related ptosis, *CSPP1* defects were reported in more than half cases (Wang et al., 2018). Besides these genes, ptosis has been described in patients carrying pathogenic variants in several other genes (Kar et al., 2018; Lee, Silhavy, Lee, et al., 2012; Shaheen et al., 2019). The Duane anomaly has been rarely reported in patients with *CEP120*‐ (Powell et al., 2020) and *KIAA0586*‐related JS (Pauli et al., 2019), while third nerve palsy has been observed in a patient with biallelic variants in *MKS1* (Bader et al., 2016).

### 3.7 Kidney

Up to 25–30% of JS individuals develop renal disease mainly presenting as juvenile NPHP, which may remain asymptomatic for several years (Fleming et al., 2017; Nuovo et al., 2020). It usually presents, in the late first or early second decade of life, as a chronic tubulointerstitial nephropathy with subtle and often unrecognized signs, such as polyuria, polydipsia, anemia, and growth failure, progressing to end‐stage renal disease (ESRD) by the end of the second decade and requiring dialysis or kidney transplantation. Pathological evaluation of the affected kidneys reveals corticomedullary cysts, atrophy, and interstitial fibrosis, whereas ultrasound evaluation may demonstrate small, scarred kidneys with increased echogenicity at the corticomedullary junction (Bachmann‐Gagescu et al., 2020; Parisi, 2019). Another form of kidney involvement consists in enlarged kidney with multiple cysts resembling autosomal recessive polycystic kidney disease (PKD) (Fleming et al., 2017; Gunay‐Aygun, 2009; Gunay‐Aygun et al., 2009).

Of the 29 individuals with kidney disease in the NIH cohort (out of 97), 31% had NPHP, 35% presented an overlapping phenotype with PKD/NPHP, 10% had a unilateral multicystic dysplastic kidney, and 24% had indeterminate‐type cystic kidney disease (Fleming et al., 2017; Vilboux, Doherty, et al., 2017). In this cohort, the gene most commonly observed to cause renal disease was *CEP290*, followed by *TMEM67* and more rarely *AHI1* (two with unilateral multicystic dysplastic kidney). Other genes identified with this phenotype included *CC2D2A, INPP5E, CEP164, NPHP1, RPGRIP1L, TMEM237*, and *TMEM216*. In particular, patients presenting NPHP associated with retinal degeneration more often had causative variants in *CEP290* and *INPP5E*, while those with NPHP but lacking retinal disease had molecular defects in *CC2D2A, TMEM67*, and *NPHP1*. Notably, seven of the 10 patients in the PKD/NPHP group had biallelic variants in *TMEM67*. This gene also causes Meckel syndrome (MKS), another ciliopathy presenting cystic kidney disease (Gunay‐Aygun, 2009; Gunay‐Aygun et al., 2009). After retinal dystrophy, renal disease was the most common associated feature reported in the UW cohort (102/407, 25%) (Bachmann‐Gagescu, Dempsey, et al., 2015). The genes mostly associated with a renal phenotype were the same as in the NIH cohort, with a significantly increased odds ratio for *CEP290* variant carriers. Similarly, we identified *CEP290* variants in 16 out of 44 probands with JS and oculorenal involvement (Brancati et al., 2007).

Several other genes have been rarely associated with renal disease in smaller reports (Alkanderi et al., 2018; Beaudin, Klein, Rouleau, & Dupre, 2017; Beck et al., 2014; Ben‐Salem et al., 2014; Chaki et al., 2012; Field et al., 2012; Huppke et al., 2015; Latour et al., 2020; Lee, Silhavy, Lee, et al., 2012; Shen et al., 2020; Slaats et al., 2016; Srour et al., 2012; Stephen et al., 2017; Thomas et al., 2014; Tuz et al., 2014; Van De Weghe et al., 2021). Among them, *RPGRIP1L* deserves a special mention, as Suzuki et al. reported 26 out of 30 mutated patients (86.7%) showing a renal phenotype. In line with these data, we searched for *RPGRIP1L* variants in 120 JS patients, detecting pathogenic alterations only in two out of 16 families, both with JS and renal involvement (~12%) (Brancati et al., 2008; Suzuki et al., 2016). Of note, no kidney disease has ever been reported in patients with pathogenic variants in *CPLANE1* or *KIAA0586*, despite these genes are among the commonest causes of JS, suggesting a negative association.

### 3.8 Liver

Approximately 10–15% of JS patients present with liver involvement, typically manifesting as congenital hepatic fibrosis (CHF) (Bachmann‐Gagescu, Dempsey, et al., 2015; Doherty et al., 2010). This incidence is likely an underestimate, as hepatic disease may manifest later in life or even remain asymptomatic in a subset of cases.

CHF results from embryonic malformation of ductal plates due to dysfunctional cilia. It is characterized by persistent embryonic ductal plate structures, progressive fibrosis of portal tracts and cystic dilation of intrahepatic biliary ducts (Desmet, 1992; Rock & McLin, 2014). Liver disease due to CHF may present with elevated serum liver enzymes (clinically asymptomatic), early onset of hepatosplenomegaly or, uncommonly in JS, more severe manifestations, such as portal hypertension, esophageal varices, and thrombocytopenia (Bachmann‐Gagescu et al., 2020; Gunay‐Aygun, 2009; Gunay–Aygun et al., 2013). As already discussed, hepatic fibrosis is often associated with ocular colobomas and sometimes with kidney disease as well. The distinctive syndromic combination of colobomas, cognitive impairment (“oligophrenia”), ataxia, cerebellar vermis hypoplasia, and hepatic fibrosis was previously referred to with the acronym “COACH” (Doherty et al., 2010). Indeed, in the UW cohort, 50 out of 362 patients (14%) had liver fibrosis, which was strongly associated to coloboma (Bachmann‐Gagescu, Dempsey, et al., 2015). Thus, the likelihood of having liver fibrosis in individuals with coloboma resulted in 6.5 times the likelihood of having liver fibrosis in individuals without coloboma.

Our research group was the first to report a strong association between liver disease and the *TMEM67* gene, detecting biallelic variants of *TMEM67* in eight of 14 (57%) JS families with congenital liver fibrosis (Brancati et al., 2009). This significant association has been subsequently confirmed (Iannicelli et al., 2010). In the NIH cohort, 21 of 22 patients (95%) with *TMEM67* variants had evidence of liver disease, accounting for half of patients with this phenotype. The significant gene‐phenotype correlation between the *TMEM67* gene and of JS with liver involvement was further confirmed in the UW cohort. Nine out of 12 (75%) *TMEM67*‐related patients presented this phenotype, with an odds ratio of 17.3 (CI 7.2–42.0). Thus, individuals with JS harboring causative variants in *TMEM67* necessitate closer monitoring to allow early diagnosis and treatment of portal hypertension. Besides *TMEM67*, other mutated genes in patients with liver disease include *CPLANE1*, *CC2D2A*, *AHI1*, and several other genes (Bachmann‐Gagescu, Dempsey, et al., 2015; Beck et al., 2014; Latour et al., 2020; Stephen et al., 2017; Strongin et al., 2018; Vilboux, Doherty, et al., 2017).

### 3.9 Skeleton

Skeletal features in JS mainly include polydactyly, which occurs in 13–15% of patients (Bachmann‐Gagescu, Dempsey, et al., 2015; Vilboux, Doherty, et al., 2017). This feature, especially when postaxial, can be found in all JS subtypes, but when occurring in combination with the MTS and oral‐facial features (e.g., tongue hamartomas, cleft lip‐palate, notched upper lip, etc.), defines a specific form of oral‐facial‐digital (OFD) syndrome known as OFD6 (Poretti et al., 2012; Valente et al., 2013) (see paragraph below).

In the UW cohort, polydactyly, which is present in 56 out of 387 (15%) of JS individuals, was significantly correlated only with biallelic variants in *CPLANE1* and *TCTN2*, but also observed in single or few patients with other gene variants (Bachmann‐Gagescu, Dempsey, et al., 2015). The NIH cohort, where polydactyly was present in 13 out of 99 JS individuals (13%), substantially confirmed the significant positive correlation between polydactyly and *CPLANE1* (Vilboux, Doherty, et al., 2017). Besides this association, polydactyly (mainly postaxial) has been described in several reports in association with the vast majority of JS known genes, including biallelic missense variants in *SUFU* (Bachmann‐Gagescu, Dempsey, et al., 2015; Bachmann‐Gagescu, Phelps, et al., 2015; Brancati et al., 2009; De Mori et al., 2017; Epting et al., 2020; Hardee et al., 2017; Kar et al., 2018; Kroes et al., 2016; Lambacher et al., 2016; Latour et al., 2020; Lee, Silhavy, Lee, et al., 2012; Powell et al., 2020; Radha Rama Devi et al., 2020; Shaheen et al., 2019; Stephen et al., 2017; Thomas et al., 2014; Van De Weghe et al., 2021; Zhang et al., 2017; Zhang, Qu, et al., 2021; Zhongling, Guoming, Yanhui, & Xiaoru, 2021).

Mild‐to‐severe scoliosis may occur in JS mainly related to hypotonia in early infancy and has been reported in about 5% of patients (Bachmann‐Gagescu, Dempsey, et al., 2015).

Interestingly, in a few families, skeletal dysplasia is present in addition to typical JS features; in particular, patients may present overlapping phenotypes of JS and Jeune asphyxiating thoracic dystrophy (JATD), a skeletal ciliopathy within the group of short rib polydactylies (Bachmann‐Gagescu, Dempsey, et al., 2015; Lehman et al., 2010). JATD patients show a long and narrow thorax with short ribs, which often lead to respiratory failure and death, as a result of an inability to fully expand the lungs; other features are short stature with undersized limbs, polydactyly, and renal cystic dysplasia. Biallelic defects in five genes have been reported identified in patients with this phenotype: *IFT172* (Halbritter et al., 2013), *CSPP1* (Tuz et al., 2014), *KIAA0586* (Malicdan et al., 2015), *CEP120* (Romani et al., 2013; Roosing, Romani, et al., 2016; Shaheen et al., 2015), and *KIAA0753* (Inskeep et al., 2022).

Other skeletal anomalies in JS are definitely rare. Among these, cited as examples, camptodactyly and bowing of long bones have been observed respectively in four and one patients out of 20 with *TMEM216*‐related JS (Valente et al., 2010). Moreover, camptodactyly of digits III and V was also present in both hands of a child with *MKS1*‐related JS (Bader et al., 2016). Other skeletal anomalies were reported in single studies, such as tibial and fibular mesomelic dysplasia in one patient with *B9D2*‐related JS (Bachmann‐Gagescu, Dempsey, et al., 2015) or abnormal cone‐shaped epiphyses of hands and feet (not associated with JATD) in one patient with biallelic variants in *CELSR2* (Vilboux, Doherty, et al., 2017; Vilboux, Malicdan, et al., 2017). Finally, club foot has been reported in a child with *RIPGRIP1L*‐related JS, even though this complication may also be present in all severe cases presenting fetal hypokinesia (Brancati et al., 2008).

### 3.10 Oral‐facial‐digital syndrome type 6

The term OFD syndromes describe a group of disorders mainly characterized by distinguishing facial features, oral abnormalities, and polydactyly. At least 18 clinical subtypes have been described, and the whole spectrum of findings tends to overlap with MKS, short‐rib thoracic dystrophies, and JS (Bruel et al., 2017).

Among these syndromes, OFD6 (also known as Varadi–Papp syndrome) has been regarded as a rare phenotypic subtype of JS. The diagnosis of OFD6 requires the MTS as well as one or more of the following features: a distinctive preaxial or mesaxial polydactyly with Y‐shaped metacarps (but the less specific postaxial polydactyly has also been observed), syndactyly and/or bifid toe, a bifid or lobulated tongue due to soft‐tissue nodules or multiple hamartomas, multiple oral frenulae, palate, and/or lip clefting, craniofacial features that include hypertelorism and upper lip notch, and hypothalamic hamartoma sometimes with absent pituitary gland (Bonnard et al., 2018; Poretti et al., 2012).

There has been some debate in the literature whether *CPLANE1* may represent the most relevant gene associated to OFD6. A first survey identified pathogenic variants in the *CPLANE1* gene in nine out of 11 fetuses with OFD6 features (82%), suggesting that this could indeed represent the major causative gene for OFD6 (Lopez et al., 2014). Other groups subsequently confirmed that *CPLANE1* is one of the causative genes for this condition (Bayram et al., 2015; Wentzensen et al., 2015; Wentzensen et al., 2016). However, we identified *CPLANE1* pathogenic variants in only two of 17 living individuals with classical OFD6 phenotype, questioning whether *CPLANE1* could be regarded as the main OFD6‐causative gene (Romani et al., 2015). In two further studies, novel *CPLANE1* recessive variants were reported in seven subjects with pure OFD6 (from five unrelated families) (Bonnard et al., 2018), as well as in four JS Chinese families with mild neurological and neuroradiological features (Liu et al., 2020). Taken together, these observations define a spectrum of phenotypes associated to *CPLANE1* variants, ranging from an exclusively neurological phenotype to the full blown OFD6 presentation. In general, features of preaxial and/or mesaxial polydactyly and hypothalamic hamartoma seem more commonly related to *CPLANE1* pathogenic variants, whereas tongue hamartomas and lingual frenula are less frequently associated with molecular defects in this gene (Lopez et al., 2014; Romani et al., 2015). Besides *CPLANE1*, several other genes have been occasionally reported to cause OFD6 (Bachmann‐Gagescu, Dempsey, et al., 2015; Ben‐Salem et al., 2014; Chevrier et al., 2016; Coene et al., 2009; Darmency‐Stamboul et al., 2013; Johnston et al., 2017; Lambacher et al., 2016; Oka et al., 2016; Shaheen et al., 2019; Thauvin‐Robinet et al., 2014; Valente et al., 2010).

### 3.11 Miscellaneous features

Although JS is not a typical dysmorphic syndrome, some facial dysmorphisms are often observed, such as prominent forehead, arched eyebrows, ptosis, trapezoid‐shaped mouth with lower lip eversion, and prognathia, which tend to change with age (Braddock et al., 2007). Nevertheless, facial features do not support the clinical diagnosis, as in many patients they can be mild or absent.

Interestingly, characteristic facial dysmorphisms (such as hypertelorism, broad, and depressed nasal bridge, frontal bossing) and macrocephaly have been evidenced in patients with biallelic hypomorphic or monoallelic truncating variants in *SUFU* as well as with biallelic variants in *KIF7* (De Mori et al., 2017; Niceta et al., 2020; Serpieri et al., 2021), suggesting these specific cranio‐facial anomalies are strictly related to abnormal activation of the Sonic Hedgehog (SHH) pathway, caused either by reduced levels (or functioning) of repressor proteins or by the constitutive activation of proteins that transduce SHH signaling (De Mori et al., 2017). Variable dysmorphic features have been reported in patients carrying pathogenic variants in several genes, without any specific association (Bachmann‐Gagescu, Dempsey, et al., 2015; Field et al., 2012; Hardee et al., 2017; Kar et al., 2018; Latour et al., 2020; Niceta et al., 2020; Stephen et al., 2017; Valente et al., 2010; Zhongling et al., 2021). Of note, all the five patients with *IFT74*‐related JS reported so far show a typical subtle cleft of the upper lip (Zhongling et al., 2021), while the three siblings with *ARMC9*‐related JS reported by Kar et al. presented joint laxity involving exclusively the metacarpophalangeal and interphalangeal joints (Kar et al., 2018).

Structural heart disease is not considered a typical JS feature, even though many ciliopathies are known to have an increased risk of cardiac malformations and their presence is of cardinal importance for the outcome. In the large UW cohort, only seven out 532 JS individuals had congenital heart defects (with a minimum prevalence of 1.3%) (Bachmann‐Gagescu, Dempsey, et al., 2015). Thus, no cardiac screening is recommended for JS patients. Yet, although the incidence of heart defects in JS is small, these have been occasionally reported in patients harboring mutations in several genes, including some prevalent ones such as *AHI1, CEP290, CPLANE1, KIAA0586*, and *MKS1* (Bader et al., 2016; Fraser & Davey, 2019; Parisi, 2009; Parisi et al., 2006; Zhang, Qu, et al., 2021). In particular, it is of interest the report of a few *CEP290* mutated cases presenting with heart defects, mainly atrio‐ventricular septal defect (Alharbi et al., 2018; Bachmann‐Gagescu, Dempsey, et al., 2015; Karp, Grosse‐Wortmann, & Bowdin, 2012; Trevino et al., 2020).

Laterality defects, such as situs inversus, frequently accompanying cardiac malformations and sometimes complicating other ciliopathies, are also rare in JS. This phenotype has been reported associated with pathogenic variants in *CC2D2A, CEP290, OFD1*, and *ZFN423* (Bachmann‐Gagescu, Dempsey, et al., 2015; Chaki et al., 2012; Parisi, 2009; Zhang, Qu, et al., 2021).

Hirschsprung's disease (manifested by intractable constipation) has been described so far in few JS individuals (Brancati et al., 2010), while this condition is significantly associated with another ciliopathy, Bardet–Biedl syndrome (Lorda‐Sanchez, Ayuso, & Ibañez, 2000).

Individuals with *OFD1*‐ and *CBY1*‐related JS have been reported to have recurrent respiratory infections similar to individuals with primary ciliary dyskinesia (Coene et al., 2009; Epting et al., 2020; Hannah et al., 2019).

Short stature and isolated growth hormone deficiency or panhypopituitarism (often associated with pituitary dysgenesis) have been observed in association to several JS genes, without clear correlations (Aljeaid, Lombardo, Witte, & Hopkin, 2019; Brancati et al., 2008; Lee, Silhavy, Zaki, et al., 2012; Lucas‐Herald et al., 2015; Sanders et al., 2015; Stephen et al., 2017; Van De Weghe et al., 2017; Vilboux, Doherty, et al., 2017; Vilboux, Malicdan, et al., 2017; Wolf et al., 2007). Micropenis and/or hypospadias have also been described in several genetic forms of JS (Bachmann‐Gagescu, Dempsey, et al., 2015; Bader et al., 2016; Hardee et al., 2017; Inskeep et al., 2022; Kroes et al., 2016; Latour et al., 2020; Lee, Silhavy, Zaki, et al., 2012; Niceta et al., 2020; Sanders et al., 2015; Valente et al., 2010; Van De Weghe et al., 2017).

The prevalence of hearing loss in the UW series was 3% (16 out of 532 JS patients); thus, higher than the general population prevalence (Bachmann‐Gagescu, Dempsey, et al., 2015; Fortnum et al., 2001). Molecular defects in several genes have been occasionally identified in patients with this complication (Bachmann‐Gagescu, Dempsey, et al., 2015; Khan et al., 2016; Shaheen et al., 2019; Tuz et al., 2014; Van De Weghe et al., 2017; Wentzensen et al., 2016).

Little is known about mortality in JS. In the UW cohort, 40 of 565 patients with JS were deceased, mainly due to extra‐neurological involvement, such as kidney disease or liver fibrosis. The underlying genetic defects had been identified in 80% (32/40) of the deceased cohort. Although the numbers were too small for any statistical analyses, six of nine individuals with biallelic variants in *CEP290* had died from complications of kidney disease, while two of the three with molecular defects in *TMEM67* had died from liver fibrosis, and all three individuals with *TCTN2*‐related JS died from respiratory complications. Thus, a close monitoring of these issues should be considered in patients with these additional risk factors (Dempsey et al., 2017).

### 3.12 Clinical and genetic overlap with other disorders

Pathogenic variants in genes that cause JS have also been identified in other ciliopathies with overlapping clinical features, such as MKS, isolated NPHP, LCA, OFDS, BBS, ACLS, JATD, and others. Shared features variably include retinal dystrophy, NPHP or cystic dysplastic kidneys, congenital liver fibrosis, polydactyly, situs inversus, occipital encephalocele, and midline oral and facial abnormalities. This marked genetic overlap, with allelism at many gene loci, is shown in Table 1.

**TABLE 1:** Genetic overlap of Joubert syndrome with other disorders

	Allelic disorders												
JS gene	MIM	ACLS	BBS	BCNS	CRD/RP	HLS	JATD	LCA	MKS	MORM	NPHP	OFDS	Reference
*ARL3*	604695				+								Holtan, Teigen, Aukrust, Bragadóttir, and Houge (2019)
*B9D1*	614144								+				Hopp et al. (2011)
*B9D2*	611951								+				Dowdle et al. (2011)
*C2CD3*	615944											+	Thauvin‐Robinet et al. (2014)
*CC2D2A*	612013								+				Mougou‐Zerelli et al. (2009)
*CEP120*	613446						+		+			+	Shaheen et al. (2015), Shaheen, Schmidts, et al. (2015), Roosing, Romani, et al. (2016)
*CEP290*	610142		+					+	+				Leitch et al. (2008), den Hollander et al. (2006), Baala et al. (2007)
*CSPP1*	611654						+		+				Tuz et al. (2014), Shaheen et al. (2014)
*HYLS1*	610693					+							Mee et al. (2005)
*INPP5E*	613037				+					+			Sangermano et al. (2021), Jacoby et al. (2009)
*IFT74*	608040		+										Lindstrand et al. (2016)
*KIAA0586*	610178					+	+						Alby et al. (2015)
*KIAA0753*	617112						+					+	Hammarsjö et al. (2017), Chevrier et al. (2016)
*KIF7*	611254	+				+							Putoux et al. (2011)
*MKS1*	609883		+						+				Leitch et al. (2008); Kyttala et al. (2006)
*NPHP1*	607100										+		Hildebrandt et al. (1997)
*OFD1*	300170				+							+	Webb et al. (2012), Ferrante et al. (2001)
*POC1B*	614784				+			+					Beck et al. (2014)
*RPGRIP1L*	610937								+				Delous et al. (2007)
*SUFU*	607035			+									Pastorino et al. (2009)
*TCTN2*	613846								+				Shaheen et al. (2011)
*TCTN3*	613847								+			+	Huppke et al. (2015), Thomas et al. (2012)
*TMEM67*	609884								+		+		Smith et al. (2006), Otto et al. (2009)
*TMEM107*	616183								+			+	Shaheen et al. (2016), Shylo, Christopher, Iglesias, Daluiski, and Weatherbee (2016)
*TMEM138*	614459								+				Lee, Silhavy, Lee, et al. (2012)
*TMEM216*	613277								+				Valente et al. (2010)
*TMEM231*	614949								+				Shaheen, Ansari, Mardawi, Alshammari, and Alkuraya (2013)
*TMEM237*	614423								+				Huang et al. (2011)
*ZNF423*	604557										+		Chaki et al. (2012)

Abbreviations: ACLS, acrocallosal syndrome; BBS, Bardet–Biedl syndrome; BCNS, basal cell nevus syndrome; CRD/RP, Cone‐rod dystrophy/retinitis pigmentosa; HLS, hydrolethalus syndrome; JATD, Jeune asphyxiating thoracic dystrophy; JS, Joubert syndrome; LCA, Leber congenital amaurosis; MKS, Meckel–Gruber syndrome; MORM: mental retardation, truncal obesity, retinal dystrophy, and micropenis; NPHP, nephronophthisis; OFDS, oral‐facial‐digital syndromes (OFDVI syndrome is not included). +: phenotype present.

The most remarkable example of this clinical and genetic overlay in primary ciliopathies is the allelism between JS and MKS, a severe malformative condition that is often lethal in utero, presenting with encephalocele and other posterior fossa anomalies, ductal plate malformation of the liver, polycystic kidneys, and polydactyly. At least 16 genes have been reported to cause both JS and MKS, and it is not uncommon to find affected siblings carrying the same biallelic variants who present either JS or MKS (Parisi, 2019; Romani et al., 2013). It still remains unclear how molecular defects in the same gene can cause such a broad phenotypic spectrum encompassing different syndromes of variable severity. However, some genotype–phenotype correlates have been established. Indeed, the near‐absence of biallelic truncating variants in some ciliopathy genes suggests that their complete loss of function is poorly tolerated in humans, leading to more severe phenotypes manifesting, such as JS with multi‐systemic involvement, MKS, some OFD syndromes, or even early fetal lethality. In support of this hypothesis, correlations between the impact of causative variant and the severity of the ciliary phenotype have been observed for some genes, such as *AHI1*, *CC2D2A*, *KIAA0753*, *MKS1*, *RPGRIP1L*, *TCTN3*, and *TMEM67*, with biallelic null variants being significantly enriched in patients with severe or lethal presentations (such as MKS, some OFD syndromes), while at least one hypomorphic missense variant being detected in milder, nonlethal conditions (such as JS, NPH, BBS, nonsyndromic retinitis pigmentosa) (Bachmann‐Gagescu et al., 2012; Ben‐Salem et al., 2014; Delous et al., 2007; Huppke et al., 2015; Iannicelli et al., 2010; Mougou‐Zerelli et al., 2009; Nguyen et al., 2017; Romani et al., 2014a; Romani et al., 2014b; Stephen et al., 2017).

## 4 CONCLUSIONS

Over the past 15 years, tremendous progress has been made in understanding the genetic causes of JS and other primary ciliopathies, and in defining the phenotypic spectrum associated with specific genes. As we are now able to diagnose JS very early in life (and even prenatally) due to recognition of the MTS, the wide availability of NGS‐based tests allowing to identify the genetic cause in up to 94% of patients has greatly improved the clinical management of young patients with JS, leading to a more accurate prognosis, adequate recommendations for surveillance of potentially affected organs and improved counseling for families.

Yet, there is still a long way to go in our understanding of this complex disorder and several aspects still remain unexplained, deeply challenging genotype–phenotype correlates. For instance, while usually complete loss of function of *RPGRIP1L* or *MKS1* causes a lethal MKS phenotype, rare biallelic truncating variants in these genes have been reported in living patients with JS (Brancati et al., 2008; Brunetti‐Pierri et al., 2021; Romani et al., 2014a; Romani et al., 2014b). Moreover, both missense and truncating variants affecting the entire length of *CPLANE1* have been found in patients with either exclusively neurological JS phenotype or severe/lethal OFD6 phenotypes (Lopez et al., 2014; Romani et al., 2015); similarly, pathogenic variants in *CEP290* and *TMEM231* have been associated to a large spectrum of ciliopathies even within the same family, without clear genotype–phenotype correlation (Coppieters, Lefever, Leroy, & De Baere, 2010; Maglic et al., 2016). Even more difficult to explain, the same pathogenic variant can be associated to a wide spectrum of phenotypes. For instance, the homozygous deletion of a 290 kb genomic region on chromosome 2 encompassing the entire *NPHP1* gene is known to cause a spectrum of phenotypes ranging from isolated NPHP to congenital OMA (although no neuroimaging data of these patients were available) to JS with or without NPHP and retinal degeneration (Betz et al., 2000; Castori et al., 2005; Parisi et al., 2004). Similarly, the same founder variant p.R73C in the *TMEM216* gene causes a JS phenotype that can be entirely neurological or variably associated to polydactyly, renal disease, oral‐facial‐digital features, and retinopathy (Valente et al., 2010).

As postulated for the BBS gene *ARL6* (Pretorius et al., 2010), a first hypothesis is that pathogenic variants in the same gene, which cause different clinical expressions, might impact different isoforms of the protein that have distinct functions. More interestingly, another possible explanation is that modifier genes or other epistatic events may play a role, supporting the concept of “mutational burden.” This term refers to the sum of all genomic variations (pathogenic and nonpathogenic), contributing to define the penetrance and expressivity of a genetic disorder. A paradigmatic example is represented by the triallelic/digenic mode of inheritance hypothesized in BBS, a ciliopathy characterized by retinal dystrophy, obesity, polydactyly, intellectual impairment, renal dysfunction, and hypogonadism. Although there are no definitive evidences, it has been suggested that autosomal recessive variants in one BBS gene may be not fully penetrant in some families, and only the co‐occurrence of a third heterozygous variant in another BBS gene eventually results in the manifestation of the disease (Zaghloul & Katsanis, 2010). This oligogenic model, with two or more genes concurring to define the final phenotype, was subsequently proposed in other ciliopathies such as NPHP (Tory et al., 2007), and increased in complexity when not only pathogenic variants but also rare variants or even common polymorphisms in other ciliary genes were found to genetically interact with the “main” recessive variants, to modulate the expressivity of the ciliary phenotype. For instance, some common heterozygous variants in the *RPGRIP1L* and *AHI1* genes were found to correlate to a higher occurrence of retinal degeneration in various ciliopathies and to an increased risk of *NPHP1*‐deleted patients to develop a more severe neurological and ophthalmological phenotype, respectively (Khanna et al., 2009; Louie et al., 2010). Thus, while recessive pathogenic variants in a causative gene likely determine much of the phenotype, it was suggested that other variants (including common ones) in modifier genes may explain a substantial portion of the observed phenotypic variability. However, this was not confirmed by an independent, systematic study, questioning the validity of the original hypothesis (Phelps et al., 2018).

The establishment of meaningful genotype–phenotype correlates, which pertains not only to JS and other primary ciliopathies but to the majority of inherited diseases, represents the greatest challenge of genetic research for the years to come; deeper phenotyping of patients, analysis of large cohorts through multicenter collaborations and a better understanding of our genomic structure and variability at the individual level will represent essential assets to successfully accomplish this task.

## CONFLICT OF INTEREST

The authors declare to have no competing financial interests.

## AUTHOR CONTRIBUTIONS

Simone Gana, and Enza Maria Valente conceived the study; Simone Gana and Valentina Serpieri wrote the manuscript; Enza Maria Valente revised the manuscript.

## Data Availability

Data sharing is not applicable to this article as no datasets were generated or analyzed during the current study.
